# Readiness to use telemonitoring in diabetes care: a cross-sectional study among Austrian practitioners

**DOI:** 10.1186/s12911-019-0746-7

**Published:** 2019-01-29

**Authors:** Domenik Muigg, Peter Kastner, Georg Duftschmid, Robert Modre-Osprian, Daniela Haluza

**Affiliations:** 1 Center for Medical Statistics, Informatics and Intelligent Systems, Institute of Medical, Information Management, Medical University of Vienna, Vienna, Austria; 2 AIT Austrian Institute of Technology GmbH, Graz, Austria; 3 Institute of Environmental Health, Center for Public Health, Medical University of Vienna, Vienna, Austria

**Keywords:** Telecare, Online survey, Austria, Diabetes mellitus, Principal components analysis, Healthcare personnel

## Abstract

**Background:**

Telemonitoring services could dramatically improve the care of diabetes patients by enhancing their quality of life while decreasing healthcare expenditures. However, the potential for implementing innovative treatment options in the Austrian public and private health system is not known yet. Thus, we analyzed the readiness to use telemonitoring in diabetes care among Austrian practitioners.

**Methods:**

We conducted an online survey among a purposive sample of Austrian practitioners (*n* = 41) using an adapted German version of the practitioner telehealth readiness assessment tool. We assessed three readiness domains for telemonitoring in the context of diabetes care, i.e. core readiness, engagement readiness, and structural readiness, and validated the German tool using principal components analysis.

**Results:**

Study subjects perceived themselves as open to innovations and also expressed optimistic attitudes towards telemonitoring in general and offering telemonitoring-based services for their patients. Participants achieved a medium average readiness level for telemonitoring (58.2, 95% CI 53.9–62.5) and were thus in a good position to use telemonitoring, although some arguments may adversely affected its use. The top three perceived benefits of telemonitoring were enhanced quality of treatment, better therapy adjustment, and reduced travel and waiting times for patients. The top three barriers were reduced personal communication, practitioner time expenditure and equally placed poor financial compensation as well as data security and privacy issues.

**Conclusion:**

Our data revealed that Austrian practitioners showed a quite moderate readiness to use telemonitoring in diabetes care. To further advance telemonitoring readiness among all pillars of diabetes care in Austria, joint efforts among healthcare stakeholders are required to overcome existing financial, organizational, and technical obstacles.

## Background

Telemonitoring is the delivery or support of health services by collecting data about health conditions through the use of information and communication technologies (ICT), while patients and healthcare providers, e.g. practitioners, hospitals, and nursing staff, are not present at the same place [1]. In 2013, the Austrian government set up the Telemonitoring Services Commission, an interdisciplinary expert committee [2]. The Commission’s advisory activities are carried out by written recommendations to the Federal Minister of Health for introducing specific telemonitoring services into Austrian standard healthcare. These services mainly address the care for the chronically ill, with diabetes being one of the most important areas of application, as about 573,000 to 645,000 people with diabetes live in Austria [3].

Diabetes treatment requires lifelong therapy with frequent medical counseling. Latest advances in diabetes care allowing for supervision of patient health parameters such as blood pressure, blood sugar, and body weight from a distance are thus a best practice example of telemonitoring-enabled self-management by chronic patients [4–7]. Disease management programs such as the landmark project “Therapy active - Diabetes Under Control” already implemented in 2007 in Austria have been shown to increase quality of care for registered type 2 diabetes patients by providing monitoring, state-of-the-art medical knowledge, and training [8]. Patients receive a monitoring set consisting of a mobile phone, a blood pressure monitor, and a blood glucose meter for home use. The measuring devices automatically forward the collected data to the practitioner. In case of measures beyond predefined limit values, the system automatically notifies the competent practitioner, who initiates appropriate actions such as drug adaptation.

Nowadays, use of basic health technologies such as a computer connected to the Internet is standard in primary care in all European countries. Also, adoption of more advanced features such as electronic and personal health records, health information exchange, and telehealth is constantly rising [9]. Given that practitioners are an important part of successful telemonitoring adoption, their respective attitudes and perceptions to barriers and benefits influence the success of national telehealth strategies [10–13].

In general, technology acceptance in the healthcare sector is steadily increasing. A recent survey showed that 60% of German citizens are open to telemonitoring [14]. Already one third could even imagine monitoring body functions via skin-implanted microchips. However, the current structural and legal frameworks hamper fast diffusion of digital services in the German healthcare market [15]. In the USA, where government-run health insurance programs are very limited, healthcare providers seem confident about the merits of telemonitoring solutions, with half of the clinics already offering such services [4].

Theoretically, the Austrian healthcare sector should be well prepared for using telemonitoring. Internet access is available almost countrywide [16, 17]. Over the next years, digital skills of the population will improve with the number of technology-savvy individuals naturally increasing [18]. However, telemonitoring is not yet available in standard Austrian medical care [19]. Also, as in other countries, the concept of telemonitoring is not well-known in the Austrian population with only 11% feeling well informed [20, 21].

Numerous diabetes telemonitoring pilot projects are currently failing in the transition to live operation, especially if they prove to be too complex and complicated for real life use [22]. The lack of sustainable business models, i.e. commercial strategies for healthcare providers offering telemonitoring services to patients, was also found to hamper adoption of telemonitoring services [23]. A better understanding of the parameters potentially influencing telemonitoring use could elucidate reasons for low acceptance and thus help to design consumer-friendly, lucrative telemonitoring solutions [24].

As little is known on the readiness, awareness, and perceptions regarding telemonitoring in the context of Austrian diabetes care, we surveyed Austrian practitioners involved in diabetes care. The two main aims of this study were the analysis of so far unstudied readiness for telemonitoring among healthcare professionals in the field of diabetes treatment and the creation and validation of a German version of the Practitioner Telehealth Readiness Assessment Tool (PRAT) [25].

## Methods

### Study design

We conducted a cross-sectional survey among a nonrandom purposive sample of Austrian practitioners involved in diabetes care. In a feasibility study approach, we developed a study questionnaire including a German version of the PRAT [25], capturing the multidimensional concept of telehealth readiness. We chose this tool due to practicability reasons, as the PRAT has been already adapted to other languages such as French-Canadian [26] and other contexts such as teledentistry [27] and is generalizable for all telehealth projects. Cross-cultural adaptions and validations allow for international comparison of study results. The PRAT is based on a scoring system that evaluates the participant’s degree of readiness for using telehealth in the three aspects core readiness, engagement readiness, and structural readiness [24–26]. Our survey tool was forward-backward translated and validated as recommended by guidelines [28–30]. The final German questionnaire was pilot-tested for appearance and content among five laypersons and five researchers. The questionnaire was adapted according to the received feedback. First, we did not offer the option “other”, because the meaning of it was unclear for the testers. Second, we used a four-point Likert scale, not the original five-point one, to avoid neutral answers limiting relevance. The German questionnaire can be obtained upon request from the authors.

### Study questionnaire

The online questionnaire consisted of 21 items. The first part of the questionnaire collected data on socio-demographic characteristics including age (in years), gender, nationality, and region, medical specialization, and statutory health insurance (yes or no). Further, we asked participants to indicate their opinion regarding telemonitoring on a 3-point scale ranging from positive to negative. We asked study subjects to rate their self-perceived level of innovativeness. Possible choices were innovator, early adopter, early majority, late majority, and laggard according to Rogers’ definitions of adopter groups in the diffusion of innovation model [31]. We were interested in current self-monitoring procedures of diabetic patients and those favored by practitioners with the choices none, handwritten (e.g. paper-based diaries), electronic (e.g. smartphone-apps, diary software, and excel lists) and various. We also asked for current involvement in the Austrian disease management program “Therapy active” [8]. We further asked for willingness to provide telemonitoring services, whether patients should cover the treatment costs, and reasons to provide these services offering the three choices improve turnover, improve service and treatment quality, and improve internal processes. We used two free-text questions to collect perceived benefits and barriers to telemonitoring.

The main part of the questionnaire consisted of the three readiness domains for telemonitoring use. Core readiness covers dissatisfaction with the status quo, expectation of change, and a need for telemonitoring services. Engagement readiness covers awareness as well as assessment of the benefits and barriers of telemonitoring. Structural readiness covers the development of adequate technical infrastructure and soft skills for telemonitoring implementation [27]. As suggested in the original PRAT, we assigned a point system to each of the three categories in order to create a score for the level of telemonitoring readiness in the context of diabetes care [26]. To obtain the score for the categories and also a global score, we summed up the means of all categories. Therefore, higher scores meant greater readiness for telemonitoring: Core readiness (3 items, Cronbach’s alpha [α] = 0.288, range 3–12 points), engagement readiness (7 items, α = 0.835, range 7–28 points), and structural readiness (7 items, α = 0.771, range 7–28 points). The total scores ranged from 17 to 68 points (17 items, α = 0.860) and where interpreted in three levels of readiness: high level: above 48, indicating that practitioners were in a good position to use telemonitoring; moderate level: between 34 and 48, indicating that certain items may adversely impact the use of telemonitoring; low level: below 34, indicating that there are barriers to the successful use of telemonitoring by practitioners.

### Data collection

German-speaking private and panel doctors working in one of the nine Austrian federal states who were directly involved in diabetes treatment, i.e. mainly general practitioners and internists specialized in endocrinology, were eligible for participation. We identified possible participants through professional networks, relevant organizations, and medical associations based on online email address lists. We sent in total 863 personalized email invitations containing a link to the questionnaire as well as two reminder emails two and four weeks after the initial contact to prompt further completions. The first page of the questionnaire was opened 60 times and fully completed 41 times (completion rate 68.3%). On average, the complete survey took 6 min (range 2:39 to 10:09 min).

The online study was open and accessible to respondents from 6 March to 2 June 2 2017 using SoSci Survey [32]. The survey included a cover letter to inform participants about the scope of the survey. Once a participant completed the survey, an electronic cookie prevented multiple submissions from the same computer. Data were stored securely and protected from unauthorized access. We did not offer any incentives for participation.

### Statistical data analysis

We conducted all statistical analyses using SPSS Version 24.0 (SPSS Inc., Chicago, IL, USA). We descriptively summarized the survey data on socio-demographic characteristics and attitudes towards telemonitoring and presented categorical data as absolute frequencies and percentages, and continuous data as mean, standard deviation (SD), and median, where appropriate. We used the bootstrapping method based on 1000 bootstrap samples to obtain 95% confidence intervals (95% CI) of observed percentages. We used α to report internal consistency of the scales and chi^2^ tests to assess subgroup differences. Further, we used principal components analysis (PCA) with varimax rotation to analyze the factor structure of the 17 items of our German survey tool, as our data met the assumptions for this procedure as used in similar studies [32, 33]. The Kaiser-Meyer-Olkin test of sampling adequacy (0.703) indicated an acceptable sample size and the Bartlett’s test of sphericity (chi^2^ 401.250, *p* < 0.001) indicated that there were correlations in our data that are appropriate for factor analysis [34]. Further, we coded and sorted responses to the free-text questions in the categories benefits and barriers for telemonitoring for subsequent qualitative content analysis [35].

## Results

The average age of participants (*n* = 41) was 51.4 years (SD 9.4, range 30 to 72 years), 56.1% were males. Most respondents were internists (70.7%), worked in a private practice (68.3%), and lived in Vienna (29.3%). Further, 43.9% of study subjects participated in the disease management program “Therapy active”. Actual self-monitoring procedures by diabetic patients and those favored by practitioners differed, especially regarding electronic diaries (19.5% vs. 29.3%). Participants were representative of the Austrian medical profession in terms of age, gender, and place of residence (data not shown) [36]. Most participants perceived themselves as early adopter (53.7%) regarding new innovations (Table 1). Participants expressed optimistic attitudes towards telemonitoring (61.0%). We found that the majority of study subjects were interested in offering telemonitoring services (agree/strongly agree: 70.8%, mean 3.0, SD 0.9). The majority of participants would ask the patients to pay for these services (agree/strongly agree: 68.3%, mean 2.8, SD 0.9), that were mostly perceived as means to increase service and treatment quality (75.6%).

**Table 1:** Practitioner attitudes towards telemonitoring

	Number	Percent
How would you rate your degree of innovativeness?^a^		
Innovator	5	12.2
Early adopter	22	53.7
Early majority	9	22.0
Late majority	2	4.9
Laggard	3	7.3
How do you feel about telemonitoring?		
Negative	6	14.7
Neutral	10	24.4
Positive	25	61.0
How would you feel about offering telemonitoring services to your patients?		
Strongly disagree	2	4.9
Disagree	10	24.4
Agree	17	41.5
Strongly agree	12	29.3
How would you feel about patients paying for telemonitoring services?		
Strongly disagree	4	9.8
Disagree	9	22.0
Agree	18	43.9
Strongly agree	10	24.4
What would you like to improve with telemonitoring services?		
Service and treatment quality	31	75.6
Internal processes	16	39.0
Turnover	9	22.0
Total	41	100.0

Note: ^a^ Item adapted from Rogers 2003 [31]

In total, 28 (68.3%) respondents added material to the free-text comments box. Quantitative context analysis revealed that the three top benefits were enhanced quality of treatment (*n* = 14, 34.1%), better therapy adjustment (*n* = 7, 17.1%), and reduced travel and waiting times (*n* = 6, 14.6%). The top three perceived barriers were reduced personal communication (*n* = 9, 22.0%), time expenditure for physicians and equally placed poor financial compensation (both: *n* = 6, 14.6%) as well as data security and privacy issues (*n* = 5, 12.2%).

Table 2 shows that in the category core readiness, the item “I have firsthand experience of the negative effects of isolation from healthcare services.” received highest scores (mean 0.24, SD 0.92). In the category engagement readiness, the item “I have the need to interact with other practitioners.” received highest scores (mean 0.24, SD 0.77). In the category structural readiness, the item “I attend to issues regarding liability and licensing when using telehealth.” received highest scores (mean 2.51, SD 1.12).

**Table 2:** Ratings of practitioner telemonitoring readiness

Telemonitoring readiness^a^	Strongly disagree		Disagree		Agree		Strongly agree		Mean	SD
	n	%	n	%	n	%	n	%		
Core readiness										
I have a feeling of dissatisfaction with the current available ways of delivering care, e.g. status quo.	8	19.5	19	46.3	9	22.0	5	12.2	2.27	0.92
I have firsthand experience of the negative effects of isolation from healthcare services (professional and educational).	2	4.9	7	17.1	11	26.8	21	51.2	3.24	0.92
I have a driving need to address a public or patient healthcare problem (as opposed to a practitioner specific one) that could be met by telemonitoring.	6	14.6	12	29.3	15	36.6	8	19.5	2.61	0.97
Engagement readiness										
I am an innovator and/or champion for telemonitoring.	10	24.4	13	31.7	12	29.3	6	14.6	2.34	1.02
I have a sense of curiosity about the influences of telemonitoring on improving the delivery of health care (potential benefits).	1	2.4	5	12.2	21	51.2	14	34.1	3.17	0.74
I have respect for others in the telemonitoring team.	5	12.2	13	31.7	13	31.7	10	24.4	2.68	0.99
I have the need to interact with other practitioners.	1	2.4	5	12.2	18	43.9	17	41.5	3.24	0.77
I have examples and evidence of telemonitoring applications in similar contexts/ environments/communities.	14	34.1	6	14.6	12	29.3	9	22.0	2.39	1.18
I communicate with other practitioners and the public concerning the benefits about telemonitoring.	13	31.7	13	31.7	9	22.0	6	14.6	2.20	1.05
I am willing to make the initial extra investment in time.	13	31.7	6	14.6	16	39.0	6	14.6	2.37	1.09
Structural readiness										
I believe telemonitoring can address scheduling concerns and apprehensions about overextended workloads.	11	26.8	13	31.7	14	34.1	3	7.3	2.22	0.94
I have 24-h access to telemonitoring equipment.	23	56.1	5	12.2	4	9.8	9	22.0	1.98	1.25
I have telemonitoring reimbursement plans in place.	31	75.6	6	14.6	2	4.9	2	4.9	1.39	0.80
I have dealt with apprehensions about the reliability in telemonitoring equipment and have good technical support and backup plans.	20	48.8	13	31.7	5	12.2	3	7.3	1.78	0.94
I have access to an established reliable and available clinical consultation network (human) when using telemonitoring.	28	68.3	4	9.8	8	19.5	1	2.4	1.56	0.90
I am provided with reliable clinical content and continuing medical education (CME) through telemonitoring.	22	53.7	15	36.6	1	2.4	3	7.3	1.63	0.86
I attend to issues regarding liability and licensing when using telemonitoring.	12	29.3	4	9.8	17	41.5	8	19.5	2.51	1.12

Note: ^a^ Items adapted from Legare 2010 [26]

### Readiness for telemonitoring

The total scores ranged from 26 to 60 points. With mean 39.6 points, the average total readiness was in the category between 34 and 48 points, according to which several points have an unfavorable influence on the use of telemonitoring by practitioners (Table 3). The mean score for core readiness was 8.1 (SD 1.8), i.e. 67.5% of maximum points. Scores for structural readiness (mean 13.1, SD 4.5, 46.8% of maximum points) were much lower than those for engagement readiness (mean 18.4, SD 4.9, 67.5% of maximum points). No statistically significant differences between readiness scores and the socio-demographic characteristics of age, gender, and medical specialization could be detected.

**Table 3:** Total and average sub-scores of practitioner telemonitoring readiness

Telemonitoring readiness^b^	Maximum points	Mean	SD	Range	% of maximum points
Core readiness	12	8.1	1.8	3–12	67.5
Engagement readiness	28	18.4	4.9	8–28	65.7
Structural readiness	28	13.1	4.5	7–24	46.8
Total	68	39.6	9.2	26–60	58.2
Telemonitoring readiness level^b^	Points	n	%	95% CI^a^	
				Upper border	Lower border
High level	> 48	8	19.5	7.3	31.7
Moderate level	34–48	20	48.8	31.7	63.4
Low level	< 34	13	31.7	17.1	48.8
Total		41	100	100	100

Note: ^a^ Based on 1000 bootstrap samples ^b^ Scores adapted from Legare 2010 [26]

The total scores are interpreted as follows: > 48: practitioners are in a good position to use telemonitoring; 34–48: certain items may adversely impact the use of telemonitoring; < 34: there are barriers to successful telemonitoring use of practitioners. While 19.5% (95% CI 7.3–31.7%) were in a good position to use telemonitoring, 31.7% (31.7–63.4%) perceived respective barriers.

### Factor analysis of the German PRAT tool

PCA identified a six factor structure explaining 78.5% of the variance with all factors loading higher than 0.4 (Table 4). We provided umbrella terms for these factors. Seven items including items from all three PRAT tool domains loaded on the first factor (motivation). Three items from structural readiness loaded on the second factor (experience), two items from structural readiness and one item from engagement readiness loaded on the third factor (resources), further two items from engagement readiness loaded on the forth factor (collaboration), whereas each of the remaining items from core readiness loaded on the fifth and sixth factor, which we named empathy and dissatisfaction, respectively.

**Table 4:** Principal components analysis for all items of the telemonitoring readiness domains core readiness (CR), engagement readiness (ER), and structural readiness (SR)

Items^a^	Factors					
	1	2	3	4	5	6
1. Motivation						
I am an innovator and/or champion for telemonitoring (ER).	0.845					
I am willing to make the initial extra investment in time (ER).	0.818					
I believe telemonitoring can address scheduling concerns and apprehensions about overextended workloads (SR).	0.720					
I communicate with other practitioners and the public concerning the benefits about telemonitoring (ER).	0.711					
I have a driving need to address a public or patient healthcare problem (as opposed to a practitioner specific one) that could be met by telemonitoring (CR).	0.699					
I have a sense of curiosity about the influences of telemonitoring on improving the delivery of health care (potential benefits) (ER).	0.569					
I have 24-h access to telemonitoring equipment (SR).	0.485					
2. Experience						
I have telemonitoring reimbursement plans in place (SR).		0.841				
I am provided with reliable clinical content and continuing medical education (CME) through telemonitoring (SR).		0.768				
I have access to an established reliable and available clinical consultation network (human) when using telemonitoring (SR).		0.677				
3. Resources						
I attend to issues regarding liability and licensing when using telemonitoring (SR).			0.781			
I have examples and evidence of telemonitoring applications in similar contexts/ environments/communities (ER).			0.778			
I have dealt with apprehensions about the reliability in telemonitoring equipment and have good technical support and backup plans (SR).			0.535			
4. Collaboration						
I have respect for others in the telemonitoring team (ER).				0.870		
I have the need to interact with other practitioners (ER).				0.719		
5. Empathy						
I have firsthand experience of the negative effects of isolation from healthcare services (professional and educational) (CR).					0.898	
6. Dissatisfaction						
I have a feeling of dissatisfaction with the current available ways of delivering care, e.g. status quo (CR).						0.915

Note: ^a^ Items adapted from Legare 2010 [26]

## Discussion

Home telemonitoring services are an appropriate and cost-effective way to manage chronic patient care [37]. Particularly in the field of diabetes, treatment takes place both in the predominantly federal state financed hospitals and in the health insurance funded outpatient sector. Integrated care therefore requires a combination of both areas, which is challenging in highly fragmented healthcare systems [38]. Moreover, telemonitoring incurs expenses in the outpatient area through more intensive care by physicians, but to a cost reduction in the inpatient area by reducing serious late effects such as foot amputations. This leads to a shift in funding and political decision-making processes.

So far, there is little evidence of the theoretical potential for telemonitoring in the Austrian healthcare sector. Some country-specific facts warrant a detailed analysis of the Austrian situation, linking diabetes and the private and panel sector. In Austria, there are significantly more private practitioners (*n* = 10,553) than panel doctors (*n* = 7208), and this trend towards privatization of medical services even increased in recent years [36]. Approximately 36% of the population is privately insured [39].

Current recommendations for the application-based implementation of telemonitoring in Austria primarily target the panel doctors sector, running long pilot phases within the health insurances [2]. These recommendations include aspects such as the economic savings potential through telemonitoring itself and should therefore not be interpreted directly for the private sector, which in some points differs substantially from the panel sector.

In the private sector, telemonitoring could be implemented faster and could offer more extensive service packages at once, assuming a disproportionate cost-benefit increase in a business model for healthcare provision [23]. Patients co-finance or even take over costs for telemonitoring for convenience reasons, practitioners usually spend more time per patient and thus have the time resources for telemonitoring, and private health insurances may co-finance or even take over costs for telemonitoring for service reasons and to increase their competitive edge. Noteworthy, a high proportion of our survey participants were private doctors, potentially reflecting their inherent interest in solving the current inconsistencies they face when offering various telemonitoring services.

A survey among Austrian healthcare experts showed that allocation of financial and technological resources were perceived as the most prominent concerns for implementing telemonitoring [13]. In our survey, the key attribute of telemonitoring, namely the place- and time independency of disease treatment and monitoring described by the benefit reduced travel and waiting times, might keep patients from physically visiting their doctors. A shift from physical to virtual doctors’ appointments would currently cause a loss of earnings due to lacking funding possibilities for telemonitoring services in the current healthcare system, potentially translating into a preference of face-to-face contacts [17]. In addition to the perception that legal regulations were not yet sufficient to offer telemonitoring services in Austria, participants also expressed privacy concerns.

It is evident that low acceptance by medical professionals hampers the widespread adoption of telemonitoring [40]. In general, the adoption of telemonitoring is poorly advanced among practitioners [41]. However, findings of a Swedish study suggest an in general positive attitude towards these applications among medical staff [42]. In our survey, participants perceived themselves as open to innovations, so that they not surprisingly also expressed optimistic attitudes about telemonitoring and offering these services to their patients, as long as they pay for these services. Still, increasing their turnover would not be the main motivation for offering these services, but rather commitment to service and facilitating of internal processes. However, providing better services for patients as well as administrative tasks are very likely to increase turnover. Despite this commercial potential, finding a suitable business model for telemonitoring seems to remain a major challenge [23]. Numerous diabetes telemonitoring pilot projects fail in the transition to live operation. For example, Chen et al. reported that patients refused a chargeable continuation of a pilot telehealth project over 18 months, despite its overall beneficial effect on their health and wellbeing [5]. Greenhalgh et al. recommended to not only learn from program failures, but also only implement technological innovations that are promising for large-scale, sustained adoption [22].

We included two free text items collecting perceived benefits and barriers of telemonitoring. The greatest advantage was seen in intensified care, i.e. enhanced quality of treatment and therapy adjustment by telemonitoring, and reduced travel and waiting times for patients. In contrast, participants feared loss of personal and empathic communication as a result of focusing more on digitally recorded vital signs than on the individual patient need. Noteworthy, participants perceived the use of telemonitoring as additional expense and not as support in the treatment of their patients: Tasks that were previously taken over by the patient would now be done by the doctor again. Doctors might see telemonitoring primarily not as a support, but rather as additional burden. Overall, the added value for health technologies might be greater for patients than for physicians. Thus, structural reforms should emphasize both on the curative thought in medical decision making and the financial aspect of a business model [43].

To address the multi-dimensionality of digital strategy and related business decisions, Thill recently suggested a tripartite division to develop a general digitization strategy for healthcare providers [44]. At the system level, the technical prerequisites must be implemented. At the patient level, practitioners need to identify the optimal digitization option beyond the scope of the system for direct patient care individually. At the management level, practitioners need to optimize the functionality of practice management in order to have a basis for digitization to further increase the agility and flexibility of practical work.

The high financial expenditures in the Austrian healthcare system range above the EU average and are partly due to structural factors such as decentralized planning and governance, fragmentation of responsibilities, and hospital-oriented healthcare provision [19]. Joint planning strategies at federal and regional level for both the public and private sector are needed. A national telemonitoring strategy should tackle the already existing social inequalities in the consumption of medical services and the potential aggravation towards a two-tier medicine [19]. Panel and private practitioners should be included in the planning of such services to increase the low readiness level shown in our study [43]. With most of respondents rating themselves as innovative, Austrian healthcare professionals seem to be in a promising mood for future innovations. So, our results did not support earlier findings that Austrian doctors are merely skeptical concerning new innovations [11–13].

To increase user friendliness as well as convertibility, telemonitoring should strive to be interchangeably usable among all healthcare stakeholders including practitioners, patients, and organizations. Currently, it seems that healthcare stakeholders work on individualized solutions without following a country-wide or even international strategy, despite the existence of a national Telehealth Services Commission [2]. Challenges that have to be addressed include adequate remuneration, predictable access to the first healthcare market, evaluation requirements, regulatory frameworks, and the lack of willingness to pay both by patients.

Our study was guided by the research question whether Austrian practitioners were ready for telemonitoring, linking a common chronic disease notably suitable for telemonitoring, i.e. diabetes, with the treating physicians working in a country with assumable adequate resources to implement these services. Our study makes an important contribution to the literature by providing a German version of the PRAT and its psychometric properties as well as validating and adapting it for assessing telemonitoring readiness. However, the findings of this study are subject to several limitations. Although the low response rate was expected in a study population of medical professionals, it limits representativeness of our study sample and generalizability of the study results [45]. Advanced recruiting strategies and incentives for participation could further increase response rates in follow-up studies [46]. Though, similar sample sizes are also reported in comparable validation studies [24, 32]. As we conducted a feasibility study, we abstained from sample size calculation. However, participants had to meet the inclusion criteria, i.e. treating diabetes patients. Thus, due to the pilot study fashion of this study, random sampling methodology was not warranted from a good scientific practice perspective. To account for the low sample size, we presented confidence interval by bootstrapping where appropriate. Also, Kaiser-Meyer-Olkin test of sampling adequacy indicated an acceptable sample size for PCA.

We used a self-perception questionnaire that focused on personal experience thus introducing survey response bias. We thus also asked participants to self-rate their degree of innovativeness using a single-item question. Nevertheless, the diffusion of innovation model was also used in related studies. As an example, Emani et al. tested this concept in the context of personal health record perception [47].

Challenges regarding translating the questionnaire from English to German included adapting the specific terminology to concepts used in the German language and confining the broader notion of telehealth to telemonitoring. Also, the use of gender-neutral language had to be ensured in the German translation. In our sample, most participants were internists and worked in a private practice, markedly meeting the attributes of the actual target population for telemonitoring business models in diabetes care. So, we considered the web-based survey as useful for conducting a survey among a hard-to-reach population of German-speaking, Internet-savvy Austrian practitioners treating diabetes patients. Also, the German version of the PRAT tool showed high internal consistency and construct validity. Due to the nature of the study, we suggest further validation of the scales and to assess the construct validity in a larger-scale study. On the basis of PCA results, the domains of the original PRAT tool should be adapted and only items with higher factor loadings should be used.

This cross-sectional study aimed at testing design feasibility for consecutive research projects. Thus, the study questionnaire could serve as a useful research instrument for assessing trends and new developments among representative study samples and evaluate progress of telemonitoring implementation among different medical fields. We suggest that several aspects could be relevant for estimating readmission of an individual doctor: digital age (digital immigrant vs. digital native), gender, medical specialization, and place of residence (rural vs. urban).

## Conclusion

The high expectations towards digitalization of healthcare provision are in contrast to the low diffusion of publicly funded telemonitoring services. To provide so far lacking evidence for prevailing perceptions and expectations in the context of the Austrian digital health economy, our study analyzed the readiness to use telemonitoring services in diabetes care. Our data revealed that Austrian practitioners, which are important stakeholders for successfully implementing a nation-wide telehealth strategy, showed a quite moderate readiness for telemonitoring. Nevertheless, participants perceived themselves as open to innovations, so that they not surprisingly also expressed optimistic attitudes about telemonitoring and offering these services to their patients. Nowadays, Austrian doctors are more open to innovations than found in recent studies, indicating a potential to enhance the availability of national telemonitoring services.

Doctors fear the loss of personal communication. Thus, we recommend establishing telemonitoring as an on-top service to the established doctor-patient-relationship instead of creating centralized institutions to treat a large number of patients. Lack of reimbursement is one of the major barriers to the widespread use of telemonitoring. Since there is a tradeoff imbalance between the inpatient and outpatient area, political organizations responsible for both areas should perform an overall cost-benefit analysis on a national level. In multisided platform business models, one side is usually subsidized to enhance overall distribution. Since we found that the value of telemonitoring is more likely on the patient side, while the workload is more on the practitioner side, doctors should be subsidized when offering telemonitoring services.

## Abbreviations

CI: Confidence interval
CR: Core readiness
ER: Engagement readiness
PCA: Principal component analysis
PRAT: Practitioner telehealth readiness assessment tool
SD: Standard deviation
SR: Structural readiness
α: Cronbach’s alpha

## References

[1] Austrian Federal Ministry of Health: Telemedicine, https://www.bmgf.gv.at/home/Gesundheit/E-Health_Elga/Telemedizin/Telemedizin (Accessed Oct 2018).

[2] Telehealth Services Commission: https://www.sozialministerium.at/site/Gesundheit/Gesundheitssystem/E_Health_ELGA/Telemedizin/Telegesundheitsdienste_Kommission_gem_auml_szlig_sect_nbsp_8_BMG. Accessed 24 Jan 2019.

[3] Griebler R, Geißler W, Winkler P: Zivilisationskrankheit Diabetes Ausprägungen-Lösungsansätze-Herausforderungen. Österreichischer Diabetesbericht 2013, Bundesministerium für Gesundheit.

[4] Young HM, Nesbitt TS (2017). Increasing the capacity of primary care through enabling technology. J Gen Intern Med.

[5] Chen L, Chuang LM, Chang CH, Wang CS, Wang IC, Chung Y, Peng HY, Chen HC, Hsu YL, Lin YS, et al. Evaluating self-management behaviors of diabetic patients in a telehealthcare program: longitudinal study over 18 months. J Med Int Res. 2013;15(12):e266. 10.2196/jmir.2699 PMC3869106 24323283

[6] Arsand E, Tufano JT, Ralston JD, Hjortdahl P. Designing mobile dietary management support technologies for people with diabetes. J Telemed Telecare. 2008;14(7):329-32. 10.1258/jtt.2008.007001 18852310

[7] Peel E, Douglas M, Lawton J. Self monitoring of blood glucose in type 2 diabetes: longitudinal qualitative study of patients' perspectives. BMJ. 2007;335. 10.1136/bmj.39302.444572.DE PMC1971180 17761996

[8] Riedl R, Robausch M, Berghold A. The evaluation of the effectiveness of Austrians disease management program in patients with type 2 diabetes mellitus - a population-based retrospective cohort study. PLoS One. 2016;11(8):e0161429. 10.1371/journal.pone.0161429 PMC4988720 27532885

[9] Codagnone C, Lupiañez-Villanueva F. Benchmarking deployment of eHealth among general practitioners. Final Report. 2013. 10.2759/24556.

[10] Jennifer LP, Kristin SV, Dawn MF, Julie CH, Paul YT, Gregory JH (2012). Health care providers style may impact acceptance of Telemonitoring. Home Health Care Manag Pract.

[11] Haluza D, Jungwirth D (2014). ICT and the future of health care: aspects of doctor-patient communication. Int J Technol Assess Health Care.

[12] Haluza D, Jungwirth D (2015). ICT and the future of health care: aspects of health promotion. Int J Med Inf.

[13] Haluza D, Jungwirth D (2016). ICT and the future of healthcare: aspects of pervasive health monitoring. Inform Health Soc Care.

[14] Rohleder B (2016). Digital health.

[15] Leppert F, Gerlach J, Ostwald DA, Greiner W (2017). Strength and weaknesses of the German digital health economy. Gesundheitswesen.

[16] Statistik Austria: Survey on ICT usage in households and by individuals, http://www.statistik.at/web_en/statistics/EnergyEnvironmentInnovationMobility/information_society/index.html (Accessed 12 Oct 2018).

[17] Muigg D, Kastner P, Modre-Osprian R, Haluza D, Duftschmid G (2018). Is Austria ready for Telemonitoring? A readiness assessment among doctors and patients in the field of diabetes. Stud Health Technol Inform.

[18] Palfrey J, Gasser U (2008). Born digital: understanding the first generation of digital natives.

[19] Hofmarcher MM, Quentin W (2013). Austria: health system review. Health Syst Transit.

[20] Haluza D, Naszay M, Stockinger A, Jungwirth D. Prevailing opinions on connected health in Austria: results from an online survey. Int J Environ Res Public Health. 2016;13(8). 10.3390/ijerph13080813 PMC4997499 27529261

[21] Chen X, Ung CO, Hu H, Liu X, Zhao J, Hu Y, Li P, Yang Q (2016). Community Pharmacists’ perceptions about pharmaceutical care of traditional medicine products: a questionnaire-based cross-sectional study in Guangzhou, China. Evid Based Complement Alternat Med.

[22] Greenhalgh T, Wherton J, Papoutsi C, Lynch J, Hughes G, A'Court C, Hinder S, Fahy N, Procter R, Shaw S. Beyond adoption: a new framework for theorizing and evaluating nonadoption, abandonment, and challenges to the scale-up, spread, and sustainability of health and care technologies. J Med Internet Res. 2017;19(11):e367. 10.2196/jmir.8775 PMC5688245 29092808

[23] Chen S, Cheng A, Mehta K (2013). A review of telemedicine business models. Telemed J E Health.

[24] Jennett P, Yeo M, Pauls M, Graham J (2003). Organizational readiness for telemedicine: implications for success and failure. J Telemed Telecare.

[25] Legare E, Vincent C, Lehoux P, Anderson D, Kairy D, Gagnon MP, Jennett P (2010). Telehealth readiness assessment tools. J Telemed Telecare.

[26] Legare E, Vincent C, Lehoux P, Anderson D, Kairy D, Gagnon M-P, Jennett P (2010). Developing and validating the French-Canadian version of the practitioner and organizational telehealth readiness assessment tools. J Telemed Telecare.

[27] Jennett P, Jackson A, Healy T, Ho K, Kazanjian A, Woollard R, Haydt S, Bates J (2003). A study of a rural community’s readiness for telehealth. J Telemed Telecare.

[28] Vallerand R. Toward a methodology for the transcultural validation of psychological questionnaires: implications for research in the French. language. 1989;30.

[29] Spiekermann C, Rudack C, Stenner M (2017). Reliability and validity of the German version of the Utrecht questionnaire for outcome assessment in aesthetic rhinoplasty (D-OAR). Eur Arch Otorhinolaryngol.

[30] Sommerfleck FA, Schneeberger EE, Orozco MC, Zamora N, Landi M, Citera G (2018). Validation and cultural adaptation of the qualisex questionnaire in patients with axial spondyloarthritis in Argentina. Rheumatol Int.

[31] Rogers EM (2003). Diffusion of innovations, vol.

[32] Nayar P, McFarland KK, Chandak A, Gupta N (2016). Readiness for Teledentistry: validation of a tool for Oral health professionals. J Med Syst.

[33] Mc Carthy VJC, Murphy A, Savage E, Hegarty J, Coffey A, Leahy-Warren P, Horgan A, O'Connell R, Marsh L, Drennan J (2018). Development and psychometric testing of the clinical leadership needs analysis (CLeeNA) instrument for nurses and midwives. J Nurs Manag.

[34] Cerny BA, Kaiser HF (1977). A study of a measure of sampling adequacy for factor-analytic correlation matrices. Multivariate Behav Res.

[35] Hsiu-Fang H, Sarah ES (2005). Three approaches to qualitative content analysis. Qual Health Res.

[36] Austrian Parliament Directorate: Development of the number of private and panel phsyiscians in Austria https://www.parlament.gv.at/PAKT/VHG/XXV/AB/AB_09691/imfname_568755.pdf (Accessed 12 Oct 2018).

[37] Darkins A, Kendall S, Edmonson E, Young M, Stressel P (2015). Reduced cost and mortality using home telehealth to promote self-management of complex chronic conditions: a retrospective matched cohort study of 4,999 veteran patients. Telemed J E Health.

[38] Duftschmid G, Dorda W, Endel G, Froschl K, Gall W, Grossmann W, Hronsky M (2012). Fragmentation of diabetes treatment in Austria - an indicator for the need for shared electronic health record systems. Stud Health Technol Inform.

[39] Association of Austrian Insurance Companies, Annual Report 2015. http://www.vvo.at/vvo/vvo.nsf/sysPages/jahresbericht.html (Accessed 12 Oct 2018).

[40] Boonstra A, Broekhuis M (2010). Barriers to the acceptance of electronic medical records by physicians from systematic review to taxonomy and interventions. BMC Health Serv Res.

[41] Codagnone C, Lupiañez-Villanueva F (2014). Benchmarking Deployment of eHealth among General Practitioners (2013)–Final Report.

[42] Gund A, Lindecrantz K, Schaufelberger M, Patel H, Sjoqvist BA (2012). Attitudes among healthcare professionals towards ICT and home follow-up in chronic heart failure care. BMC Med Inform Decis Mak.

[43] Hannan TJ, Celia C (2013). Are doctors the structural weakness in the e-health building?. Intern Med J.

[44] Thill K-D: Digitalisierung der Arztpraxis: Wie man mit der Transformations-Trias zur Umsetzungs-Strategie gelangt. IFABS: BENCHMARK! 2017.

[45] Sadler GR, Lee H-C, Seung-Hwan Lim R, Fullerton J (2010). Recruiting hard-to-reach United States population sub-groups via adaptations of snowball sampling strategy. Nurs Health Sci.

[46] Baltar F, Brunet I (2012). Social research 2.0: virtual snowball sampling method using Facebook. Internet Res.

[47] Emani S, Yamin CK, Peters E, Karson AS, Lipsitz SR, Wald JS, Williams DH, Bates DW. Patient perceptions of a personal health record: a test of the diffusion of innovation model. J Med Internet Res. 2012;14(6):e150. 10.2196/jmir.2278 PMC3517342 23128775

